# Natalizumab alters the TCR repertoire after one year of treatment in four MS patients

**DOI:** 10.1186/1479-5876-9-S2-P26

**Published:** 2011-11-23

**Authors:** Laure Michel, Marion Salou, Alexandra Garcia, Nicolas Degauque, Anne Salmon, Annie Elong Ngono, Arnaud Nicot, Sandrine Wiertlewski, Jean-Paul Soulillou, Sophie Brouard, David Laplaud

**Affiliations:** 1 INSERM U643, Nantes University, Nantes, France; 2 Neurology Dept., Nantes Hospital, Nantes, France

## Background

Natalizumab is an approved drug used in relapsing and aggressive Multiple Sclerosis (MS). This is a monoclonal anti-integrin antibody that decreases the transmigration of immune cells through the blood-brain-barrier in animal models of MS. In human, the number of T cells in the CSF is dramatically decreased while the number of circulating lymphocytes usually increases. We hypothesized that in patients treated with natalizumab, the infiltration of T cells in the brain parenchyma decreases. These cells may accumulate in the blood of the patients and thus alter the T cell repertoire.

## Objectives

We assessed whether the treatment by natalizumab resulted in the apparition of Vβ clonal selections one year after the beginning of the treatment.

## Methods

The T Cell Receptor (TCR) repertoire was investigated by the analysis of the CDR3 length distribution of the β chain. The blood of MS patients was sampled just before the beginning of Natalizumab, and six months and one year after. CDR3 spectratyping was performed at those three different time points. Each profile was assessed as “gaussian”, “altered polyclonal”, “altered oligoclonal” or “altered monoclonal” and the alterations observed were compared.

## Results

Four relapsing-remitting MS patients were included in this study. At baseline, we observed more alterations in the CD8^+^ compartment compared with the CD4^+^ one. One year after the beginning of the treatment, the global percentage of alterations did not increase for CD4^+^ or CD8^+^ cells. However, for three patients, we observed the apparition of private Vβ clonal selections.

## Discussion/perspectives

Given the mechanisms of action of natalizumab, one may hypothesize that the clonal cells observed in the blood after one year of treatment could have a major role in the pathophysiological process in the disease. We now plan to analyze the phenotype and the reactivity of these clones against myelin antigens.

